# How to Manage Patients with Lenalidomide-Refractory Multiple Myeloma

**DOI:** 10.3390/cancers15010155

**Published:** 2022-12-27

**Authors:** Felipe de Arriba de la Fuente, Carmen Montes Gaisán, Javier de la Rubia Comos

**Affiliations:** 1 Haematology and Medical Oncology Department, Hospital General Universitario Morales Meseguer, Instituto Murciano de Investigación Biomédica (IMIB)-Arrixaca, Universidad de Murcia, 30008 Murcia, Spain; 2 Haematology Department, Hospital Universitario Marqués de Valdecilla, 39008 Cantabria, Spain; 3 Haematology Department, Hospital Universitario y Politécnico La Fe and Universidad Católica “San Vicente Mártir”, CIBERONC CB16/12/00284, 46001 Valencia, Spain

**Keywords:** multiple myeloma, lenalidomide, relapse, refractory

## Abstract

**Simple Summary:**

Lenalidomide is considered a key drug in the treatment of patients with multiple myeloma (MM), an incurable haematological cancer. In fact, most of the regimens used to treat early stage MM patients contain lenalidomide. This extensive use, however, has now led to treatment resistance. In this review, we explore the different options available to these patients, summarise the current knowledge and explore potential future treatments.

**Abstract:**

Although lenalidomide-based combinations, such as lenalidomide plus a proteasome inhibitor or an anti-CD38 monoclonal antibody, improve the overall response rate, progression-free survival, and overall survival of patients with relapsed/refractory multiple myeloma (RRMM), there is a tendency to use these regimens as a frontline treatment. This strategy has led to the development of refractoriness early in the disease course, usually after the patient’s first treatment. Since lenalidomide-free regimens have so far shown limited efficacy in lenalidomide-refractory patients, there is an unmet need for other treatment options. In this review, we discuss the therapeutic options available to treat the general population of lenalidomide-refractory patients (mono, double and triple refractory) and the subpopulation of patients with other high-risk features such as renal failure, extramedullary disease, and high-risk cytogenetics. Moreover, new promising individual therapies and the possible impact of immunotherapy in RRMM patients are debated.

## 1. Introduction

Multiple myeloma (MM), a haematological cancer characterised by uncontrolled proliferation of monoclonal plasma cells in the bone marrow [1], accounts for approximately 13% of all haematological malignancies and 1% of all cancers [2]. Although MM remains incurable, we have witnessed outstanding advances in the management of patients with relapsed/refractory MM (RRMM) in the past 15 years. The availability of numerous novel classes of drugs (Table 1) that are mostly used in combination [3,4,5], has substantially improved overall response rate (ORR), progression-free survival (PFS), overall survival (OS), and minimal residual disease (MRD). However, most patients eventually relapse or become refractory to treatment, and median PFS (mPFS) in many cases is less than 12 months [6].

Patients with RRMM not previously treated with lenalidomide may benefit from new highly active lenalidomide-based triplet combinations, such as carfilzomib plus lenalidomide and dexamethasone (KRd) [7], daratumumab plus lenalidomide and dexamethasone (DaraRd) [8], ixazomib plus lenalidomide and dexamethasone (IRd) [9] or elotuzumab plus lenalidomide and dexamethasone (EloRd) [10,11,12]. Unfortunately, due to the extensive use of frontline lenalidomide-based therapies, in both young (as post-transplant maintenance) and elderly patients (DaraRd, bortezomib plus lenalidomide and dexamethasone (VRd)), lenalidomide refractoriness early in the disease course is increasingly common, and rules out the use of lenalidomide-based regimens in subsequent lines [4,13]. Refractoriness is defined as no response to primary therapy (lenalidomide in our case) or progression within 60 days of the last dose [14]. Consequently, there is now a pressing need to develop lenalidomide-sparing regimens for RRMM.

Until recently, typical lenalidomide-sparing regimens available for clinical use in RRMM included carfilzomib plus dexamethasone (Kd) [15] or daratumumab plus bortezomib and dexamethasone (DaraVd) [16], however, both have limited efficacy in the lenalidomide-refractory population [17]. Recent evidence has shown the combination of pomalidomide plus bortezomib and dexamethasone (PVd) to be superior to bortezomib plus dexamethasone alone (Vd) in the management of patients with lenalidomide-refractory RRMM, although PFS was lower compared to lenalidomide-sensitive patients [18].

There is, therefore, a pressing need for effective lenalidomide-free regimens to overcome the limitations of currently available combinations for lenalidomide-refractory patients. In this review, we explore the remaining unmet needs in the treatment of RRMM resulting from the scarcity of effective lenalidomide-free combinations and assess the current therapeutic options.

**Table 1:** Available regimens for patients with RRMM.

Family	Active Compound	Combination	Therapeutic Indication ^a^	Ref.
**Proteasome** **inhibitors (PI)**	Bortezomib (V)	VRd	Not approved for RRMM	[19]
		Vd	Adult patients with progressing MM who have received at least one previous regimen and have undergone HPCT or are not candidates for HPCT	[20]
		VdT-PACE	Not approved for RRMM	[21]
	Carfilzomib (K)	KRd	Adult patients with MM who have received at least one previous regimen	[7,19]
		Kd	Adult patients with MM who have received at least one previous regimen	[15]
		KPd	Not approved for RRMM	[19,22,23]
		KCd	Not approved for RRMM	[24]
	Ixazomib (I)	IRd	Adult patients with MM who have received at least one previous regimen	[9,19,25]
		IPd	Not approved for RRMM	[19]
**Immuno-** **modulatory agents (IMiD)**	Iberdomide (cereblon E3 ligase modulators (CELMoDs))	-	Not approved for RRMM	[26]
	Lenalidomide (R)	Rd	Adult patients with MM who have received at least one previous regimen	[8,27,28]
	Pomalidomide (P)	PVd	Adult patients with MM who have received at least one previous regimen (including lenalidomide)	[18,29]
		Pd	Adult patients with RRMM who have received at least two previous regimens (including lenalidomide and bortezomib) and who have progressed on the latest therapy	[30,31,32]
		PCd	Not approved for RRMM	[33]
	Thalidomide (T)	VdT-PACE	Not approved for RRMM	[21]
**Anti- CD38 MoAb**	Daratumumab (Dara)	DaraRd	Adult patients with MM who have received at least one previous regimen	[8,19,34]
		DaraVd	Adult patients with MM who have received at least one previous regimen	[16,20]
		DaraPd	In RRMM patients after disease has not improved with lenalidomide and PI or when treated with them separately	[19,35]
		DaraKd	Adult patients with MM who have received at least one previous regimen	[36]
		DaraCd	Not approved for RRMM	[37]
	Isatuximab (Isa)	IsaKd	Adult patients with MM who have received at least one previous regimen	[38,39]
		IsaPd	Adult patients with RRMM who have received at least two previous regimens (including lenalidomide and PI) and who have shown disease progression on the last therapy	[40]
**Anti SLAMF7 monoclonal** **antibody**	Elotuzumab (Elo)	EloRd	Adult patients with MM who have received at least one previous regimen	[10,11,12,41]
		EloPd	Adult patients with RRMM who have received at least two previous treatments (including lenalidomide and PI) and who have shown disease progression on the last therapy	[42]
**Antibody-drug conjugate (ADC)**	Belantamab mafodotin (belamaf) ^b^	Bela monotherapy	Adult patients with MM who have received at least four prior therapies and whose illness is refractory to PI, IMiD and anti-CD38 MoAb, and who have shown disease progression on the last therapy	[43,44]
**Bi-specific drugs (BiABs)**	Teclistamab	-	Conditional marketing authorisation for adult patients with RRMM, who have received at least three prior therapies	[45,46]
**CAR T-cell**	Idecabtagene vicleucel (ide-cel)	-	Conditional marketing authorisation for adult patients with RRMM, who have received at least three prior therapies, including an IMiD, a PI and an anti-CD38 MoAb and whose disease has worsened since the last treatment	[47]
	Ciltacabtagene autoleucel (cilta-cel)	-	Conditional marketing authorisation for adult patients with RRMM, who have received at least three prior therapies, including an IMiD, a PI and an anti-CD38 MoAb and whose disease has worsened since the last treatment	[48]
**BCL-2 inhibitor**	Venetoclax	-	Not approved for RRMM	[49]
**Selective** **inhibitor of** **nuclear** **export**	Selinexor (S)	SVd	Adult patients with MM who have received at least one previous regimen	[50]
		Sd	Adult patients with MM who have received at least four previous therapies, whose illness is refractory to at least two PIs, two IMiDs and one anti-CD38 MoAb and has worsened since the last treatment	[51]
**Alkylating agents**	Carmustine	Dexa-BEAM	Secondary therapy and conditioning treatment prior to ASCT in malignant haematological diseases (non-Hodgkin’s lymphoma and Hodgkin’s diseases)	[52]
	Cisplatin	VdT-PACE	Not approved for RRMM	[21]
		PACE	Not approved for RRMM	[53]
		DCEP	Not approved for RRMM	[54]
		Dexa-BEAM	Not approved for RRMM	[52]
		VdT-PACE	Not approved for RRMM	[21]
		KCd	Not approved for RRMM	[24]
	Melphalan (M)	DaraMPV	Not approved for RRMM	[55]
	Bendamustine	-	Not approved for RRMM	[56]
**Anti-inflammatory steroids**	Dexamethasone (d)	VdT-PACE	Not approved for RRMM	[21]
**Macrolide** **antibiotic**	Clarithromycin (Cla)	ClaPd	Not approved for RRMM	[57]
**Alkaloid**	Etoposide €	VdT-PACE	Not approved for RRMM	[21]
**Cytotoxic** **antibiotics**	Doxorubicin (A)	VdT-PACE	Not approved for RRMM	[21]
**Antimetabolites**	Cytarabine	Dexa-BEAM	Not approved for RRMM	[52]

^a^ According to their summary of product characteristics (EMA); ^b^ in process of withdrawal of the US marketing authorisation. ASCT: autologous stem cell transplantation; ClaPd: clarithromycin pomalidomide and dexamethasone; DaraCd: daratumumab plus cyclophosphamide and dexamethasone; DaraKd: daratumumab plus carfilzomib and dexamethasone; DaraMPV: daratumumab plus melphalan, pomalidomide and bortezomib; DaraPd: daratumumab plus pomalidomide and dexamethasone; DaraVd: daratumumab plus bortezomib and dexamethasone; DaraRd: daratumumab plus lenalidomide and dexamethasone; DCEP: dexamethasone, cyclophosphamide, etoposide, and cisplatin; Dexa-BEAM: dexamethasone, carmustine, cytarabine, etoposide, and melphalan; EloPd: elotuzumab plus pomalidomide and dexamethasone; EloRd: elotuzumab plus lenalidomide and dexamethasone; HPCT: hematopoietic progenitor-cell transplantation; IPd: ixazomib plus pomalidomide and dexamethasone; IRd: ixazomib plus lenalidomide and dexamethasone; IsaKd: isatuximab plus carfilzomib and dexamethasone; IsaPd: isatuximab plus pomalidomide and dexamethasone; KCd: carfilzomib plus cyclophosphamide and dexamethasone; Kd: carfilzomib plus dexamethasone; KPd: carfilzomib plus pomalidomide and dexamethasone; KRd: carfilzomib plus lenalidomide and dexamethasone; PACE: cisplatin, doxorubicin, cyclophosphamide, and etoposide; PVd: pomalidomide plus bortezomib and dexamethasone; Rd: lenalidomide and dexamethasone; RRMM: relapsed/refractory multiple myeloma; Sd: selinexor and dexamethasone; SVd: selinexor plus bortezomib and dexamethasone; VdT-PACE: bortezomib plus dexamethasone and thalidomide-cisplatin, doxorubicin, cyclophosphamide, and etoposide.

## 2. Lenalidomide-Refractory Patients

Since the introduction of lenalidomide for the treatment of RRMM patients [27,28,58], its use as induction or maintenance therapy in newly diagnosed MM has been rising [5,59]. As lenalidomide is often administered until disease progression, lenalidomide refractoriness at relapse has become increasingly common [60]. Patients with lenalidomide-refractory MM are a heterogeneous population for which a universal regimen is not yet available.

It is likely that most lenalidomide-refractory patients progress during post autologous stem cell transplantation (ASCT) maintenance context after having been previously treated with bortezomib. However, a substantial number will not become bortezomib-refractory. Therefore, alternative regimens for this patient population include different combinations of a monoclonal antibody (MoAb), a PI, or pomalidomide.

Three trials have tested the combination of an anti-CD38 MoAb, a PI, and dexamethasone in patients with RRMM: the CASTOR, CANDOR and IKEMA trials.

The CASTOR trial analysed the combination DaraVd vs. Vd [20,61]. The number of lenalidomide-refractory patients was low in the DaraVd group (24%), and the analysis of this subgroup showed that the DaraVd group displayed a poor mPFS of 7.8 months (vs. 4.9 months in the Vd group) [20].

The isatuximab plus carfilzomib and dexamethasone (IsaKd) regimen was evaluated in the IKEMA study [39,62,63], where 32% of IsaKd and 34% of Kd-treated patients were lenalidomide-refractory. After a median follow-up of 44 months (*n* = 49 [27.4%] remaining in the IsaKd arm and *n* = 11 [8.9%] in the Kd group), mPFS was 35.7 months in the IsaKd group, in contrast with the 19.2 months in the control group [62]. ORR in IsaKd-treated patients was slightly higher than in Kd-treated patients (87% vs. 83%), and more obvious improvements were observed in very good partial response or better (VGPR, 73% vs. 56%) [39]. Complete response (CR) (44.1% vs. 28.5%), and MRD negativity rates (33.5% vs. 15.4%) were favoured by the addition of isatuximab [62]. A recent subgroup analysis showed similar results. IsaKd-treated patients who were refractory to lenalidomide (most of whom were <65 years old) showed a better PFS (PFS hazard ratio (HR) = 0.6), VGPR (66.7% vs. 35.7%), MRD negativity (24.6% vs. 9.5%), and CR rates (38.6% vs. 11.9%) than Kd-treated patients, and their PFS-event-free probability at 18 months was 53% vs. 31% [64]. Based on these results, the authors concluded that IsaKd should be used as the standard of care (SOC) for RRMM patients.

The daratumumab plus carfilzomib and dexamethasone (DaraKd) regimen analysed in the CANDOR trial improved mPFS compared to Kd (28.6 vs. 15.2 months) and Global Health Status/Quality of Life (GHS/QoL) score in RRMM patients with one to three prior lines of treatment [36,65,66]. Although only 32% of patients in the CANDOR trial were lenalidomide-refractory, pre-planned subgroup analysis of this subset and *post hoc* analysis found that mPFS in the DaraKd group rose to 28.1 months, while it was reduced to 11.1 months in the Kd group [36]; the ORR of these patients was 79.8%, showing efficacy and safety results consistent with the overall benefit of DaraKd over Kd [67].

Regarding combinations with pomalidomide, the PVd regimen poses another alternative. It showed an mPFS of 11.20 months in the OPTIMMISM phase 3 clinical trial that included with 70% lenalidomide-refractory and 13% bortezomib-refractory population [18]. However, mPFS in the lenalidomide refractory population was less than 10 months (9.5 vs. 5.6 months). In this case, the frequency of some grade 3 and 4 adverse events in the PVd-treated arm should be considered when assessing its suitability.

**Figure 1:**
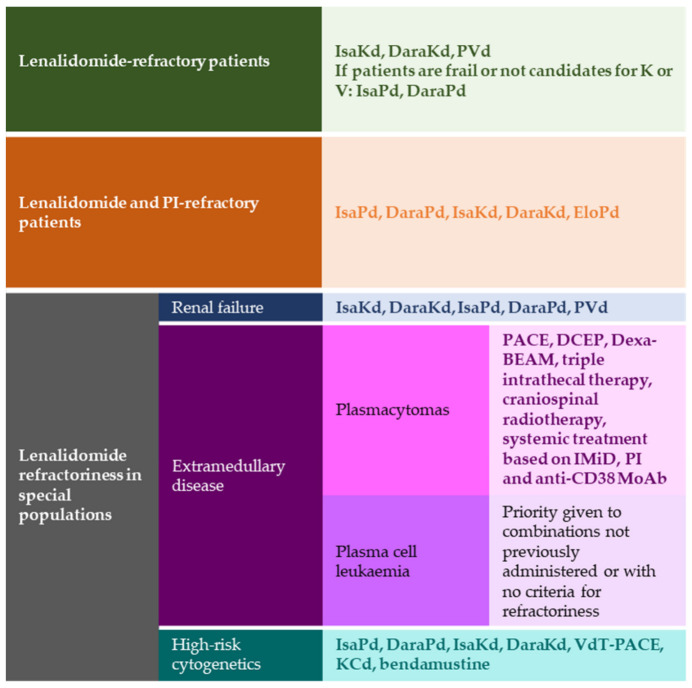
Available treatments for lenalidomide-refractory MM. Dara: daratumumab; DaraKd: daratumumab plus carfilzomib and dexamethasone; DaraMPV: daratumumab plus melphalan, pomalidomide and bortezomib; DaraPd: daratumumab plus pomalidomide and dexamethasone; DaraVd: daratumumab plus bortezomib and dexamethasone; DaraRd: daratumumab plus lenalidomide and dexamethasone; DCEP: dexamethasone, cyclophosphamide, etoposide, and cisplatin; Dexa-BEAM: dexamethasone, carmustine, cytarabine, etoposide, and melphalan; EloPd: elotuzumab plus pomalidomide and dexamethasone; EloPVd: elotuzumab plus pomalidomide, bortezomib and dexamethasone; IMiD: immunomodulatory drugs; Isa: isatuximab; IsaKd: isatuximab plus carfilzomib and dexamethasone; IsaPd: isatuximab plus pomalidomide and dexamethasone; KCd: carfilzomib plus cyclophosphamide and dexamethasone; Kd: carfilzomib plus dexamethasone; KPd: carfilzomib plus pomalidomide and dexamethasone; PACE: cisplatin, doxorubicin, cyclophosphamide, and etoposide; PI: protease inhibitor; PVd: pomalidomide plus bortezomib and dexamethasone; VdT-PACE: bortezomib plus dexamethasone and thalidomide-cisplatin, doxorubicin, cyclophosphamide, and etoposide.

Overall, these results suggest that combinations that include an anti-CD38 MoAb plus carfilzomib are among the regimens that achieve better results in the lenalidomide-refractory population Anti-CD38 MoAb plus bortezomib combinations have shown poorer results, compared to the above-mentioned combinations (Figure 1).

Transplant-ineligible patients (usually, patients ≥70 years old and/or with comorbidities [2,68]) relapsing while on lenalidomide treatment could benefit from anti-CD38 MoAb and Kd or Pd regimens (Figure 1).

In the IKEMA study, half the population were aged ≥65 years, and the addition of isatuximab to their treatment prolonged mPFS (mPFS was not reached, while in the control group was 17.18) [39]. PFS HR was 0.43 (reduced to 0.24 in ≥75 years old) [39]. The subgroup analysis of patients aged >70 years (refractoriness to lenalidomide 34.6–52.9%) showed that VGPR, CR and MDR values were higher in the IsaKd group compared to the Kd group (73.1 vs. 55.9, 38.5 vs. 23.5, 23.1 vs. 11.8, respectively), and a better depth of response. Moreover, VGPR and CR were similar to those obtained in the IsaKd-treated group of patients aged <70 years (73.1 vs. 72.4 and 38.5 vs. 40.2, respectively). In addition, definitive discontinuation because of treatment-emergent adverse events was lower in the IsaKd group (11.8%) than in Kd-treated patients (23.5%) [69].

Over 65-year-old patients represented 48–50% of the population in the CANDOR study. Patients treated with the DaraKd scheme presented improved mPFS compared to the Kd-treated group (25.9 vs. 15.3) and their PFS HR was 0.73 [65].

Regarding combinations with Pd, in the ICARIA trial (isatuximab plus pomalidomide and dexamethasone (IsaPd) vs. Pd, 94% of lenalidomide-refractory patients), the population was initially stratified to <75 years and ≥75 years. IsaPd-treated patients aged ≥75 years and 65–74 years showed longer mPFS (11.4 and 11.6 vs. 4.5 and 8.6 months, respectively), and ORR (53.1–64.7% vs. 31–38.9%) than those in the Pd arm in a subgroup analysis [70]. The VGPR or better rate observed was three-fold higher in patients aged ≥75 years treated with IsaPd than in their Pd-treated counterparts (31.2% vs. 0%) and increased almost two-fold in patients aged 65–74 years (32.3% vs. 13%). Moreover, values were similar to those in the <65 years group. In addition, health-related quality of life was higher in the ≥75 years group than in the 65–75 years group [70]. A subgroup analysis of lenalidomide-refractory patients showed a similar improvement in PFS (11.4 vs. 5.6 months) and ORR (59% vs. 31.4%), regardless of age [71].

Lastly, in the APOLLO trial (daratumumab plus pomalidomide and dexamethasone (DaraPd) vs. Pd), the age cut-off was set at 65 years old. Patients treated with DaraPd showed an mPFS of 14.2 months vs. 7 months in the Pd group [35]. Remarkably, this population had received a median of two prior lines of treatment, while patients in the ICARIA trial had received three [70] and the percentage of refractoriness to lenalidomide was lower [35,40,70,72].

The efficacy observed with the anti-CD38 and Kd combinations positions them as SOC for patients refractory to lenalidomide. However, combinations with Pd after the first relapse may represent a good option in patients with cardiovascular risk or other pathologies and may reduce the number of hospital visits.

## 3. Multirefractory Patients

### 3.1. Double Refractory Patients

Bortezomib, like lenalidomide, has been the backbone of nearly all induction and maintenance regimens following ASCT. As a result, most patients will have been exposed to bortezomib at the time of relapse, and some are likely to be bortezomib-refractory. Potential alternative therapies for these patients include the combination of an anti-CD38 MoAb plus pomalidomide (IsaPd, DaraPd and elotuzumab with pomalidomide and dexamethasone (EloPd)). IsaPd was investigated in the ICARIA-MM trial [40,71], in which every patient had received at least two prior lines of treatment. Nearly all (94%) of patients were lenalidomide-refractory (60% on last line) and mPFS in the IsaPd arm was 11.4 months vs. 5.59 in the Pd group [40,71]. Double refractory patients (71%) treated with IsaPd had an mPFS of 11.2 months (similar to the mPFS achieved in the intention-to-treat population treated with IsaPd), whereas mPFS in Pd-treated patients was only 4.8 months; PFS HR was 0.60 [71]. ORR was around 59% in lenalidomide (early and last line) and double lenalidomide/PI-refractory [71].

The DaraPd combination was weighed against Pd in the APOLLO trial [35], in which 42% of patients were refractory to lenalidomide and PI. Results showed that the DaraPd regimen prolonged mPFS by 5.5 months compared to Pd (12.4 vs. 6.9 months; PFS HR = 0.63). ORR, VGPR or better, and negative status for MRD also improved (69% vs. 46%; 51% vs. 20%; and 9% vs. 2%). After treatment with DaraPd, the median PFS was 9.9 months in the patients refractory to immunomodulatory agents and PI vs. 6.5 months in those treated with Pd (PFS HR = 0.66).

In the EQUULEUS (MMY1001) clinical trial [73], which also studied the safety and tolerability of DaraPd, in patients with RRMM with ≥2 prior lines, 89% of patients were lenalidomide-refractory, whereas 71% were double refractory to PI and immunomodulatory agents (IMiD). Median PFS was 8.8 months, mOS was 17.5 months, and ORR was 60% [73]. In a retrospective study that analysed the safety and efficacy of DaraPd in RRMM patients, all patients were lenalidomide-refractory, and 91% were bortezomib-refractory [74]. This study reported significant benefits for the DaraPd treatment in daratumumab and pomalidomide *naïve* patients (mPFS was not reached within 41 months of follow-up) [74]. Despite these promising results, the retrospective cohort only included 12 patients and results need to be confirmed in larger, prospective trials.

In the phase 2 ELOQUENT-3 trial, the EloPd combination [42] achieved an mPFS of 10.3 months, with an ORR of 53%. Moreover, the EloPd group showed a higher VGPR or better than Pd-treated patients (20% vs. 9%). In this study, elotuzumab-treated patients were 90% lenalidomide-refractory and 68% double refractory to lenalidomide and PI. The PFS HR for those who were double refractory resulted in 0.56; overall PFS HR was 0.51.

These trials showcased the potential of anti-CD38 plus pomalidomide not only for lenalidomide-refractory patients but also for patients double refractory to lenalidomide and a PI. Despite their similar results in mPFS, the difference in their percentage of refractoriness and the number of previous lines of treatment should be considered when evaluating potential treatments (Figure 1).

### 3.2. Triple Refractory Patients

Current treatment of MM is based on starting the most effective regimens as soon as possible to obtain a deep, sustained response, instead of reserving these therapies for an eventual relapse [75,76]. This drug combination strategy explains the growing number of cases that meet the criteria for refractoriness to a PI, an IMiD, and an anti-CD38 MoAb even in the early stages of the disease. These triple refractory patients pose a new challenge because there is as yet no SOC defined. Even though daratumumab and isatuximab have shown significant PFS improvements in RRMM patients in several clinical trials, when the disease develops resistance to any anti-CD38 MoAb, the alternatives for novel therapy outside of clinical trials are still limited.

Refractoriness to lenalidomide, PI and anti-CD38 MoAb in the early stages of MM is a recent situation that has not been explored specifically in clinical trials. Similar to other conditions, and given the lack of evidence, the decision to treat in these cases is determined by the patient’s situation and comorbidities, prior therapies, and any restrictions the centre may have on obtaining certain drug combinations. A valid option is to avoid administering lenalidomide and anti-CD38 MoAb in favour of regimens such as Kd (especially in patients with good overall status and no cardiovascular risk factors), which achieved an mPFS of 15.2 months in a study that included 36% of lenalidomide-refractory patients, or PVd [36,38]. To improve these results, three studies added cyclophosphamide (KCd [24], phase 2) or pomalidomide (KPd [22,23], phase 1-1/2) to the combination. Although these regimens have not yet been approved, they have shown an mPFS of 20.7 months in the KCd trial [24] and 7.2 months [22] and 10.3 months [23] in the two trials with the KPd combination. Noteworthy, all patients in the KPd phase 1 trial were lenalidomide-refractory.

The retrospective multicentre MAMMOTH study [77] evaluated the prognosis of MM patients who met the criteria for refractoriness to an anti-CD38 MoAb alone or in combination as part of the last line of treatment. The prognosis for each group of patients worsened as refractoriness increased, with a median OS of 8.6 months. The highest median OS (11.2 months) was observed in non-triple refractory patients. The scheme carfilzomib plus an alkylating agent stood out among the different treatments used after progression with an ORR of 47.4%, followed by PACE-like combinations (45.8%), alkylating agents (44.4%), daratumumab plus IMiD (36.6%) and carfilzomib (32.4%). In non-triple refractory patients, other factors aside from refractoriness to an anti-CD38 MoAb affected prognosis. These patients had received a median of three prior lines, usually including lenalidomide (94.7%), and a significant proportion of patients were refractory to lenalidomide, pomalidomide or bortezomib (43.9%, 40.4% and 22.8%, respectively), a circumstance that undoubtedly influenced the outcome of salvage therapy [77].

The recently published LOCOMMOTION study [78,79] included a series of real-world patients receiving ≥3 lines of treatment that included more than 80 different regimens: 94.4% were refractory to an IMiD, 91.9% to an anti-CD38 MoAb, and 79.4% to a PI. In total, 73.8% were triple-class refractory [79]. Similar to the MAMMOTH study, outcomes after SOC in this group of patients were poor, with an ORR of 29.8%, a median PFS of 4.6 months, and a median OS of 12.4 months. Only 0.4% of the patients achieved CR (*n* = 1) [79].

Due to the potential synergy achieved with the association of an anti-CD38 MoAb with IMiD, other combinations based on re-treatment with an anti-CD38 MoAb have also been explored. In this context, the combination of an anti-CD38 MoAb with an IMiD was able to induce a response in anti-CD38 MoAb-refractory patients, especially when anti-CD38 MoAb refractoriness was a result of its use in monotherapy compared to its administration in combination (PFS: 5.1 months vs. 1.9 months, respectively), in the MAMMOTH study [77]. However, this effect was not observed when the anti-CD38 was associated with a PI. This finding was confirmed in a retrospective study of patients with lenalidomide, pomalidomide, and daratumumab refractoriness criteria, in which the DaraPd combination achieved a PR or better in 33.3% of patients and disease stabilisation in 50% [74]. In short, these data suggest that the DaraPd regimen should be considered a good salvage treatment option in patients who are refractory to lenalidomide and daratumumab, especially if refractoriness to the anti-CD38 MoAb is a consequence of its use in monotherapy.

Noteworthy, daratumumab and isatuximab MoAb have different binding epitopes on the CD38 molecule, and therefore different biological effects [80]. This characteristic could justify the approaches that evaluate crossing the use of these drugs when refractoriness to one of them is found. Indeed, isatuximab in monotherapy has been found to achieve disease stabilisation in daratumumab-refractory patients in advanced stages [81,82,83,84,85]. Nevertheless, a recent analysis of the ICARIA trial shows that the immediate use of an anti-CD38 monoclonal antibody with currently available combinations appears to be less effective after receiving IsaPd, leading to a median PFS of 2.2 months [86]. Therefore, we need additional data before recommending the administration of anti-CD38-based therapy after a previous regimen, including one of these antibodies.

In summary, the administration of carfilzomib or pomalidomide may be appropriate for RRMM patients (Figure 1). Data from the LOCOMMOTION and MAMMOTH studies highlighted the poor outcomes observed in RRMM patients using existing SOC [79]. In this scenario, immunotherapy with new drugs such as belantamab, chimeric antigen receptor (CAR)-T cell therapies, or administration of inhibitors such as venetoclax or selinexor will probably pave the way to better treatment strategies (Section 3.2.1).

#### 3.2.1. New Therapies

Several new agents are in the advanced stages of clinical research. Regulatory agencies have already licenced several of these due to their ability to induce a response in patients refractory to one or more classes of drugs routinely used in MM.

B-cell maturation antigen (BCMA), present in myeloma cells, is an ideal target for developing targeted therapies for MM [45]. Belantamab mafodotin (belamaf), a monomethyl auristatin F-conjugated anti-BCMA MoAb, was used as monotherapy in the DREAMM-2 clinical trial in patients with triple refractory MM [43]. After comparing two doses of belamaf, a duration of response of 11 months and an OS of 13.7 months were achieved. Clinical trials currently underway are exploring belamaf in combination with other agents, in which the drug is used at lower or more widely spaced doses. Bi-specific monoclonal antibodies (BiAbs) and CAR-T cells have been initially tested in late-stage MM with excellent results [87,88,89], justifying the design of the first clinical trials for early stage MM, particularly in patients with high-risk features. BiAbs are molecules that facilitate tumour lysis by binding to two antigens, one present on the tumour cell and the other on the T lymphocyte. These drugs have shown their ability to induce quality responses in patients with MM in advanced stages of the disease, with a favourable safety profile and could be used in combination with other anti-myeloma therapies [45]. Recently, teclistamab, a BiAb binding CD3+ T cells and BCMA, received conditional EMA approval based on a phase 1–2 study performed by Moreau et al. [46]. The authors showed that after 14.1 months of follow-up, mPFS was 11.3, ORR was 63%, and 39.4% of patients achieved CR or better in a population in which 77.6% were triple refractory. CAR-T cells are produced by modifying the patient’s autologous T cells to express a chimeric antigen receptor on their surface that targets an antigen present on the surface of the tumour cell. Despite their significant limitations to their widespread use, such as high cost and a complex, time-consuming production process (which delays administration for several weeks), they can rapidly induce quality responses in heavily treated MM. A meta-analysis evaluating this therapy in 640 patients with MM in advanced stages obtained an 80.5% OR and 44.8% complete remissions [90]. The PFS with this therapy is 12.2 months. Although outcomes surpassed expectations in this patient population, these data indicate that most patients progress after CAR-T therapy [90,91]. Idecabtagene vicleucel (idecel) is a CAR T-cell therapy that has been shown to induce responses in the majority of MMRR patients who had received at least three prior lines of treatment, achieving MRD-negative status in 26% of treated patients [47]. Based on this data, idecel was the first CAR T-cell therapy to gain FDA and EMA approval for use in MM. Recently released data show that the safety and 30-day responses in a real-world setting (ORR: 83%, CR: 34%) were comparable to the clinical trial population [92]. In the same line, the CARTITUDE-1 study, which included heavily pre-treated RRMM patients, has demonstrated that a single infusion of ciltacabtagene autoleucel (cilta-cel), a CAR-T featuring two BCMA-targeting single domain antibodies, resulted in deep and durable responses, with 98% of patients responding to therapy. Notably, 80% of patients who responded achieved a stringent complete response [48]. This interesting data provided the basis for its recent FDA and EMA conditional approvals.

Finally, other studies have evaluated small molecules, such as selinexor or venetoclax. Selinexor (S) is an inhibitor of the nuclear export protein XPO-1 that forces the accumulation of tumour suppressor proteins at the nuclear level [93]. Its use in combination was evaluated in a phase 2 clinical trial in penta-refractory MM patients and achieved an ORR of 26%, duration of response of 4.4 months, and a median OS of 8.6 months [51]. It is now being tested in earlier stages [50] or combined with conventional MM treatment regimens [93]. Venetoclax is a potent and selective oral BCL-2 inhibitor already used in various haematological malignancies [49].

Venetoclax with Vd was compared to placebo with Vd in the BELLINI phase 3 clinical trial [49], and its benefit was limited to patients with t(11;14) or high BCL2 expression. Although cases with PI refractoriness criteria were specifically excluded from this trial, other combinations are being tested that could be useful for multi-refractory patients [93]. Finally, the new IMiD-derived class of cereblon E3 ligase modulators (CELMoDs), a family of oral agents that include iberdomide, avadomide, mezigdomide (CC-92480), and CC-885 [94], are currently in the early stages of clinical research for the treatment of advanced-stage MM, but have already demonstrated their ability to overcome resistance to IMiD and the possibility of use in combination with other anti-myeloma agents to seek a synergistic effect [95].

## 4. Lenalidomide Relapse in Special Populations

### 4.1. Renal Failure

Renal dysfunction may be present in 20–50% of patients with MM [96]. Although most MM treatments have no contraindication in patients with renal impairment (RI), patients with severe renal insufficiency were not eligible to participate in most of the clinical trials that led to the indication of the various drug different combinations for the management of RRMM. Furthermore, some of these studies showed that mild or moderate renal dysfunction had a detrimental effect on PFS [20,97]. Nevertheless, the management of relapse in lenalidomide-refractory patients with renal dysfunction does not differ from the strategies used in patients with normal renal function. The treatment of choice will rely on safe and fast-acting drugs that do not need major dose adjustments [76], such as pomalidomide, carfilzomib, bortezomib, daratumumab, and isatuximab [98]. Therefore, PVd, DaraKd, IsaKd, DaraPd, and IsaPd, are safe in patients with renal failure, including those on dialysis [63,99,100,101,102] (Figure 1). However, caution is advised when prescribing other drugs whose safety data in renal failure are not yet available.

### 4.2. Extramedullary Disease

Plasmacytoma is defined as a monoclonal proliferation of plasma cells forming a tumour mass, either by contiguity from a bone lesion (paraskeletal plasmacytomas) or by haematogenous dissemination of neoplastic cells with exclusive involvement of soft tissues (extramedullary plasmacytomas) [103]. The lack of a uniform criterion to define plasmacytoma and specific imaging techniques available for its correct evaluation has led to a certain amount of confusion in establishing the incidence of this disease, its prognostic impact, and the most appropriate management [103,104]. Nevertheless, the presence of extramedullary plasmacytomas, even at the time of initial diagnosis, makes MM a very high-risk entity. There are no prospective clinical trials in patients with the extramedullary disease, and data on the efficacy of different anti-myeloma agents are limited and not always consistent, which explains the lack of a defined SOC for this group of patients. In the context of relapsed patients, and whether or not they are lenalidomide refractory, consensus guidelines recommend using in first-line chemotherapy regimens similar to those used in patients with lymphoma (PACE: cisplatin, doxorubicin, cyclophosphamide, and etoposide; DCEP: dexamethasone, cyclophosphamide, etoposide, and cisplatin; Dexa-BEAM [52]: dexamethasone, carmustine, cytarabine, etoposide, and melphalan). PIs can also be considered a valid option in patients that have not previously received this type of therapy or have achieved a prolonged response [103]. A *post hoc* analysis of the IKEMA trial observed an increase in PFS HR (0.574), response rate (50.0% vs. 28.6%), CR (33.3% vs. 14.3%), and VGPR (33.3% vs. 14.3%) in the group of patients with soft-tissue plasmacytomas that had been treated with IsaKd (*n* = 12) vs. those receiving Kd (*n* = 7) [105]. Median PFS in IsaKd-treated patients was 18.76 months, while in Kd-treated patients, it was not calculable.

In this regard, a subgroup analysis of patients from the ICARIA trial with soft-tissue plasmacytomas (7.8% of patients, *n =* 24) showed that IsaPd improved mPFS (4.57 vs. 1.56 months) and ORR (50% vs. 10% responders) compared to Pd [106]. Half of all patients will achieve some, albeit short, response with these treatments; therefore, intensification therapy such as haematopoietic progenitor transplantation should be attempted in responders whenever possible (Figure 1).

Plasma cell leukaemia (PCL) is an extremely aggressive disease. Under new diagnostic criteria, PCL has been defined as the identification of >5% plasma cells on peripheral blood differential white cell count [107]. According to this, PCL can be divided into primary PCL—when the leukemic phase is present at the time of diagnosis—and secondary PCL—when it arises as part of the relapse or progression of pre-existing MM. Patients with PCL are excluded from most clinical trials, so few clinical data are available to guide their management, which is even more obvious in salvage therapy. In the absence of evidence, treatment strategy for relapse of primary or secondary PCL generally involves active combinations in patients with MM, especially if they have not received them previously or have not shown refractoriness to them [108].

### 4.3. High-Risk Cytogenetics

There is currently no regimen specifically indicated for RRMM patients with high-risk cytogenetics. Abnormalities comprise del(17p), t(4;14), t(14;16) and most recently, gain/amp(1q21) [76]. Comparisons between clinical trials are challenging due to the different cut-off values selected to determine whether the cytogenetic characteristics of an individual are considered high risk. In some studies, these cut-off values are as high as ≥50% for del(17p) or ≥30% for t(4;14) or t(14;16) (ICARIA and IKEMA clinical trials [39,109]), but others use lower values, such as ≥5% for del(17p) or ≥3% for t(4;14) or t(14;16) (TOURMALINE-MM1 [9]), or do not report them (for example, in the case of the APOLLO or CANDOR trials [35,36]). Drug combinations are similar to those indicated for the general population (Figure 1), but experts are aware that the duration and depth of response will usually be inferior in the high cytogenetic risk group [76].

The subgroup analysis of the ICARIA-MM study showed that ORR and VGPR were similar to those observed in standard-risk cytogenetic patients in the IsaPd arm (50% vs. 65% and 29.2% vs. 32%, respectively). In addition, IsaPd-treated patients in the high-risk cytogenetic group showed better results than their Pd counterparts; their mPFS was prolonged compared to Pd (7.5 vs. 3.7 months; PFS HR = 0.66) [109]. The subgroup analysis of the IKEMA study included patients with del(17p), t(4;14), t(14;16) and gain/amp(1q21). PFS HR in the IsaKd arm in both groups (high risk and standard risk cytogenetics) was improved vs. Kd (0.724 and 0.440, respectively) [110]. Interestingly, patients with t(4;14) exhibited better response than those with del(17p), and those with gain/amp(1q21) alone vs. those with gain/amp(1q21) and del(17p)/t(4;14)/t(14;16). In addition, CR, VGPR and MRD tended to improve more in IsaKd-treated patients with gain/amp(1q21) than in those with the other chromosomal abnormalities [110]. A recently published subgroup analysis of patients with gain/amp(1q21) showed that mPFS and mOS improved in patients with gain/amp(1q21) treated with IsaPd/IsaKd vs. Pd/Kd, and that the addition of isatuximab in patients with and without gain/amp(1q21) gave similar results, at least for 11 months [111]. Depth of response was improved in different subgroups of 1q21 (1q21+, isolated 1q21+, gain [1q21], and amp [1q21]) after treatment with isatuximab) [111]. Regarding combinations with daratumumab, results show that mPFS was extended with the DaraKd combination in the population with high cytogenetic risk (mPFS = 15.6 vs. 5.6 months; PFS HR = 0.49); in the standard risk population, it was not calculable [65]. DaraVd treatment also improved mPFS compared to Vd, but it remained poorer than in the standard-risk population (11.2 vs. 7.2 months in high-risk and 19.6 vs. 7.0; PFS HR = 0.45 vs. 0.26). However, ORR in high-risk and standard-risk cytogenetics were similar [61]. The DaraPd regimen improved mPFS in 1.8 months in patients with high-risk cytogenetics compared to the Pd combination (5.8 vs. 4 months; PFS HR = 0.85) [35].

Different strategies have been suggested to overcome the poor prognosis associated with high-risk cytogenetics, but there is scant evidence of their usefulness. These include the use of more intense regimens, with combinations of four drugs with different mechanisms of action (e.g., DaraKPd), or the incorporation of conventional cytostatic agents, such as cyclophosphamide (e.g., KCd [24]) or bendamustine [76]). In patients who relapse rapidly and aggressively, a treatment regimen based on conventional chemotherapy (e.g., VdT-PACE [21]) may be beneficial [76]. In very selected cases, allogeneic haematopoietic stem cell transplantation (HSCT) can be considered [112], although this is a very high-risk procedure and is decreasingly being indicated in the management of MM.

New drugs that have achieved excellent results in MM patients with advanced disease have appeared recently (Section 3.2.1). CAR-T therapy, BiAbs, and conjugated monoclonal antibodies, among others, are postulated as the most promising therapies to provide more robust control of the disease in risk situations. The first studies in early stage patients have already been launched.

## 5. Discussion and Expert Opinion

The growing number of lenalidomide-refractory patients at the time of relapse has boosted the development of several combinations of drugs for this patient population. However, the decision-making process remains challenging for most physicians due to the small number of patients in some of these studies, the short follow-up, and the absence of head-to-head comparisons.

The number of patients that receive anti-CD38 MoAb in the first relapse or even in the first line is increasing. Patients that have received lenalidomide in the first line (in monotherapy or triplets with bortezomib (not necessarily PI-refractory)) and progress while on lenalidomide would not a priori be candidates for a lenalidomide-based combination but could benefit from different treatments of choice comprising carfilzomib (DaraKd or IsaKd) or pomalidomide (DaraPd or IsaPd). Although still valid, regimens without an anti-CD38 MoAb, such as Kd, KCyd or PVd, have less promising results.

Double refractory patients have a poor prognosis, particularly if they are young and have risk factors. In these cases, combinations with an anti-CD38 MoAb and carfilzomib or pomalidomide would be suitable. Other options without an anti-CD38 MoAb include Pd, PCyd, KPd, or polychemotherapy as a bridge to cellular therapy.

In combination with new drugs, early treatment conditions progressively increase multirefractory cases in the early phases. There is no SOC for these patients, and the outcomes are still poor. New drugs with new mechanisms of action have shown efficacy in monotherapy or in association with corticoids and are likely to be used in combination. New immunotherapies, including CAR-T, conjugated antibodies, or bi-specific engager therapies, have marked a turning point in treating patients with RRMM.

Despite the small number of patients and the short follow-up, these approaches will play a pivotal role in the treatment of MM in the near future. We believe that the design of clinical trials that analyse these therapies in earlier phases of the disease, taking advantage of the patient’s better-preserved immune system, will have far-reaching implications. Although relapses cannot be ruled out, immunotherapies achieve excellent responses in the advanced stages of the illness, and their increased use in the early phases will probably further improve outcomes. However, it must be acknowledged that only a minority of these new drugs are currently available for clinical use, and access will vary across countries due to the potential for worsening financial toxicity. Therefore, until these new drugs enter clinical practice, conventional drugs, such as IMiD, PI, and anti-CD38 MoAb, will remain the cornerstone of MM treatment in most patients.

There are still unmet medical needs in different patient subgroups. No studies have specifically addressed the management of lenalidomide-refractory patients with special conditions. Although RI is one of the most common complications of symptomatic MM, most trials in patients with RRMM exclude patients with severe RI. Despite substantial improvements in survival among these patients following the introduction of novel anti-myeloma agents, RI remains one of the most challenging situations in clinical practice. Most treatments are active and do not need dose adjustment in patients with RI, even those requiring renal dialysis. Some drugs to improve patient management are under development, but caution is advised.

Extramedullary disease is a hazardous condition in RRMM, and regimens that can induce a rapid response are required. Cytotoxic-containing therapies are often chosen for treatment.

Finally, careful analysis of cytogenetic subgroups in trials comparing different treatments remains an important goal, and cross-trial comparisons may provide insight into the effect of new drugs in this context. However, experts need to agree on the definition of analytical techniques, the proportion of abnormal cells, and the recommended treatment regimens. If the disease is extremely aggressive, other regimens that include classic cytotoxic drugs that induce rapid responses should be considered. These patients are also good candidates to test new treatments, such as CAR-T o BiAbs, which are effective in the advanced stages of the disease.

Different ways to further improve clinical outcomes could be optimising the available drug combinations, including proper sequence of successive regimens, and reconsidering the duration of treatment. In this regard, comparative studies will help define treatment strategies that extend clinical benefit for patients with RRMM and re-evaluating the current continuous treatment approach in every patient may also help reduce the emergence of resistant clones and potentially improve prognosis.

## 6. Conclusions and Future Perspective

In light of this promising therapeutic armamentarium for lenalidomide-refractory RRMM, one of the scientific community’s most important tasks will be to determine the ideal drug combination early in the disease course to maximise survival and minimise adverse consequences. Current data support the use of anti-CD38 MoAb in combination with Kd or Pd in lenalidomide-refractory patients after their first relapse. Nonetheless, clinical trials focused on the lenalidomide-refractory population are needed. Together, these therapies should lead to higher response rates, a more durable duration of response, less toxicity, and prolonged survival, making MM an increasingly treatable disease.
